# Dissolved organic phosphorus bond-class utilization by *Synechococcus*

**DOI:** 10.1093/femsec/fiae099

**Published:** 2024-07-13

**Authors:** Emily M Waggoner, Kahina Djaoudi, Julia M Diaz, Solange Duhamel

**Affiliations:** Department of Molecular and Cellular Biology, University of Arizona, 1007 East Lowell Street, Tucson, Arizona, AZ 85721, United States; Department of Molecular and Cellular Biology, University of Arizona, 1007 East Lowell Street, Tucson, Arizona, AZ 85721, United States; Geosciences Research Division, Scripps Institution of Oceanography, University of California, San Diego, La Jolla, CA 92093, United States; Department of Molecular and Cellular Biology, University of Arizona, 1007 East Lowell Street, Tucson, Arizona, AZ 85721, United States

**Keywords:** alkaline phosphatase, dissolved organic phosphorus, phosphoanhydride, phosphoester, phosphonate, *Synechococcus*

## Abstract

Dissolved organic phosphorus (DOP) contains compounds with phosphoester, phosphoanhydride, and phosphorus–carbon bonds. While DOP holds significant nutritional value for marine microorganisms, the bioavailability of each bond-class to the widespread cyanobacterium *Synechococcus* remains largely unknown. This study evaluates bond-class specific DOP utilization by *Synechococcus* strains from open and coastal oceans. Both strains exhibited comparable growth rates when provided phosphate, a phosphoanhydride [3-polyphosphate and 45-polyphosphate], or a DOP compound with both phosphoanhydride and phosphoester bonds (adenosine 5′-triphosphate). Growth rates on phosphoesters [glucose-6-phosphate, adenosine 5′-monophosphate, bis(4-methylumbelliferyl) phosphate] were variable, and neither strain grew on selected phosphorus–carbon compounds. Both strains hydrolyzed 3-polyphosphate, then adenosine 5′-triphosphate, and lastly adenosine 5′-monophosphate, exhibiting preferential enzymatic hydrolysis of phosphoanhydride bonds. The strains’ exoproteomes contained phosphorus hydrolases, which combined with enhanced cell-free hydrolysis of 3-polyphosphate and adenosine 5′-triphosphate under phosphate deficiency, suggests active mineralization of phosphoanhydride bonds by these exoproteins. *Synechococcus* alkaline phosphatases presented broad substrate specificities, including activity toward the phosphoanhydride 3-polyphosphate, with varying affinities between strains. Collectively, these findings underscore the potentially significant role of compounds with phosphoanhydride bonds in *Synechococcus* phosphorus nutrition and highlight varied growth and enzymatic responses to molecular diversity within DOP bond-classes, thereby expanding our understanding of microbially mediated DOP cycling in marine ecosystems.

## Teaser

Globally abundant open ocean and coastal ocean *Synechococcus* strains preferentially utilize phosphoanhydrides over phosphoesters.

## Introduction

The picocyanobacterium *Synechococcus* is an abundant photosynthesizer, inhabiting multiple climate zones, as well as open ocean and coastal regions (Palenik et al. 2006, Zwirglmaier et al. 2008, Tai and Palenik 2009, Sohm et al. 2016, Bock et al. 2018, Nagarkar et al. 2021). Phosphorus (P) availability is a dominant factor influencing *Synechococcus* ecophysiology and abundance, as phosphate (P*i*) can be present at biologically low, colimiting, and limiting concentrations in surface mixed-layer oligotrophic regions (Krom et al. 2010, Lomas et al. 2010, Kretz et al. 2015, Djaoudi et al. 2018, Sosa et al. 2019, Yuan et al. 2024). As one strategy to cope with P*i* scarcity, marine microorganisms, including *Synechococcus*, use dissolved organic phosphorus (DOP), which typically constitutes the dominant fraction of the dissolved P pool in open ocean surface waters (Lomas et al. 2010, Duhamel et al. 2014, 2021, Karl and Björkman 2015, Ranjit et al. 2024). Even in P*i*-replete regions, the labile fraction of DOP is rapidly recycled (Benitez-Nelson and Buesseler 1999, Nausch et al. 2018), emphasizing the role of DOP in microbial P nutrition and its potentially significant role in sustaining primary productivity (Björkman et al. 2018, Whitney and Lomas 2019, Duhamel et al. 2021, Letscher et al. 2022).

Natural marine DOP can be classified into three P bond-classes: phosphoesters (P-esters), polyphosphates (PolyP), and phosphonates (Phn) (Kolowith et al. 2001, Young and Ingall 2010). P-esters (+V oxidation state), typically in the form of monoesters (P–O–C) and diesters (C–O–P–O–C), are the most abundant (∼80%–85% of the high molecular weight dissolved organic matter) (Young and Ingall 2010). P-ester bioavailability has historically focused on monoesters (Moore et al. 2005, Wang et al. 2016, Filella et al. 2022), though there is increasing support that certain marine species can utilize diesters (Yamaguchi et al. 2005, Hull and Ruttenberg 2022). PolyP is a polymer composed of orthophosphate repeating units linked by phosphoanhydride (P–O–P) bonds and is estimated to account for ∼8%–13% of the high molecular weight dissolved organic matter (Young and Ingall 2010, Diaz et al. 2016, Saad et al. 2016). While PolyP can be found in organic and inorganic forms, it is typically measured in the organic P pools as it requires prior hydrolysis to yield soluble reactive P (Armstrong et al. 1966, Karl and Tien 1992, Karl and Björkman 2015). Although poorly characterized, PolyP quantifications revealed similar relative concentrations in the North Pacific Subtropical Gyre (Diaz et al. 2008, 2016) and Indian Ocean (Martin et al. 2018) surface waters. Phosphonates (P–C) include P in its +III oxidation state and account for ∼5%–10% of the high molecular weight dissolved organic matter (Young and Ingall 2010). Naturally and artificially occurring phosphonates have the potential to be bioavailable P sources for certain marine bacteria (Repeta et al. 2016, Sosa et al. 2019), marine cyanobacteria (Ilikchyan et al. 2009, 2010), and marine eukaryotic taxa (Wang et al. 2016, Whitney and Lomas 2019). Despite the importance of DOP, the relative bioavailability of specific bond-class compounds is poorly resolved (Karl and Björkman 2015, Diaz et al. 2016, Granzow et al. 2021).

To acquire P*i* from DOP, marine microorganisms can use P-hydrolases, including alkaline phosphatases (AP), which are regulated by the Pho Regulon, itself controlled by P*i* availability (Cembella et al. 1984, Duhamel et al. 2010, 2014, Santos-Beneit 2015, Huang et al. 2018, Li et al. 2019, Sisma-Ventura and Rahav 2019). APs include isoforms (phoA, phoD, and phoX) that are widely distributed in prokaryotes, including *Synechococcus* (Tetu et al. 2009, Cox and Saito 2013), and are known for hydrolyzing P-monoesters, and possibly P-diesters (Huang et al. 2018, Srivastava et al. 2021). The enzymes responsible for PolyP degradation are not well-characterized, though there is increasing evidence that marine APs may be able to hydrolyze PolyP (Martin et al. 2018, Lin et al. 2019, Adams et al. 2022). Certain coastal *Synechocococus* strains, namely CC9311 and CC9902, lack the Pho Regulon. This absence has been hypothesized to be an adaptation to their growth in P-replete environments (Su et al. 2007). The lack of this regulatory complex contrasts with its identification in both open ocean WH8102 and coastal WH5701 *Synechococcus* strains (Su et al. 2007, Scanlan et al. 2009, Tetu et al. 2009, Christie-Oleza et al. 2015, Santos-Beneit 2015). The Pho Regulon also controls the expression of the *phn* operon, a multigene complex that allows for the transport and use of phosphonates (Kamat and Raushel 2013, Tiwari et al. 2015, Stosiek et al. 2020). The *phn* operon encodes a range of P-C cleaving enzymes, including the P–C lyase complex, which supports the hydrolysis of a range of phosphonates. Additional enzymes, such as phosphonohydrolases, act on phosphonates independently of P*i* availability and are present in a range of prokaryotes (McGrath et al. 1997, Benitez-Nelson et al. 2004, Quinn et al. 2007, Villarreal-Chiu et al. 2012).

Though P-esters are thought to dominate microbial DOP nutrition, recent studies using culture isolates indicate that to some microbial groups, PolyP plays an important role (Lin et al. 2016, Diaz et al. 2018, 2019, Duhamel et al. 2021, Adams et al. 2022). Specifically, picocyanobacteria strains *Prochlorococcus* MED4, MIT9312, and MIT9313, and *Synechococcus* WH8102 can grow on short-chain 3-polyphosphate (3-PolyP) as a sole source of P (Moore et al. 2005). For marine bacteria cultures of *Ruegeria pomeroyi* DSS-3, PolyP and P-ester substrates can support equivalent growth (Adams et al. 2022), while diatom cultures of the genus *Thalassiosira* exhibit preferential degradation of PolyP over P-esters (Lin et al. 2016, Diaz et al. 2018, 2019). Considering the widespread presence of *Synechococcus* and its importance in biogeochemical cycling, we assessed bond-specific bioavailability and utilization of DOP compounds to open ocean (WH8102) and coastal (WH5701) *Synechococcu*s strains. We hypothesized that *Synechococcus* can hydrolyze both P-anhydrides and P-esters, possibly using AP, supporting a flexible P metabolism favoring their wide distribution across global surface waters.

## Materials and methods

### Synechococcus growth, axenicity, and cell counts

Axenic *Synechococcus* WH8102 (open ocean strain) and WH5701 (coastal strain) were obtained from the National Center for Marine Algae and Microbiota (NCMA, Bigelow Laboratories, East Boothbay, Maine). Both strains were grown in batch culture using SN media (Waterbury et al. 1986) made with aged, filtered (0.2 µm), and autoclaved (120°C, 30 min) seawater from station ALOHA (A Long-term Oligotrophic Habitat Assessment). At the late-exponential phase, cultures were transferred in triplicate to one of two SN media: (1) +P*i* (45 µmol l^−1^ KH_2_PO_4_; following Waterbury et al. 1986) and (2) −P*i* (no KH_2_PO_4_ added; P*i* below detection limit). All cultures were incubated at 25°C on a 12 h:12 h light cycle at 130 µmol m^−1^ s^−1^ in sterile culture flasks with a vent cap (0.22 µm hydrophobic membrane). *In vivo* fluorescence (IVF) was measured (AquaFluor®, Turner Designs) as a proxy for *Synechococcus* biomass. An aliquot of all culture treatments was inoculated in Luria-Bertani (LB, Miller) broth once per growth phase and incubated in the dark at 25°C for 3 days to verify that the cultures remained axenic during each experiment. A culture was considered axenic if the absorbance, measured at 610 nm, did not increase significantly over this time, which was the case for all samples. Over the growth curve, *Synechococcus* culture aliquots were collected, fixed (final concentration of 0.2% paraformaldehyde), and stored at −80°C until cell abundance analysis using the Guava® EasyCyte flow cytometer (Millipore). Briefly, *Synechococcus* was enumerated in unstained samples based on red fluorescence (i.e. chlorophyll) and forward scatter signals using a low flow rate of 0.24 µl s^−1^ for 1 min. Instrument-specific beads (Guava® Check Kit, Luminex) were used to calibrate the instrument.

### Growth on DOP substrates

The capacity of *Synechococcus* WH8102 and WH5701 to grow on different DOP bond-classes as a sole P source was tested in −P*i* SN media amended with a single DOP substrate (45 µmol l^−1^ P, final concentration; Waterbury et al. 1986). To examine *Synechococcus* growth across DOP bond-classes, two P-monoesters, one P-diester, two P-anhydrides, and four phosphonates were selected as representatives of the DOP pool. Additionally, a P-monoester containing P-anhydride bonds was selected to examine growth on a DOP compound containing multiple bond-classes. Specifically, representative DOP compounds included the P-monoesters glucose-6-phosphate (Glc-6-P) and adenosine 5′-monophosphate (AMP); the P-diester bis(4-methylumbelliferyl) phosphate (BisMUF-P); the short and long chain polyphosphates: 3-PolyP and 45-polyphosphate (45-PolyP); the P-monoester and P-anhydride containing adenosine 5′-triphosphate (ATP); and the phosphonates: 4-nitrophenyl phenylphosphonate (4-NpPn), 2-aminomethylphosphonic acid (2-AEPn), methylphosphonic acid (MPn), and ethylphosphonic acid (EPn). Two separate experiments were carried out in triplicate to test growth on (1) P-monoester and PolyP substrates, and (2) a P-diester and phosphonates. Two control treatments (culture grown in +P*i* and −P*i* media) were carried out in triplicate for each experiment. IVF was measured daily in each treatment over ∼20 days, and cell axenicity was tested every ∼5 days. Growth rates were calculated as the slope of the best-fit line over the natural log-linear portion of the IVF curve (typically within days 0–7, except for AMP which was within days 7–13 to account for the delayed growth; Table S1).

To address the possibility of abiotic degradation of the amended DOP substrates under culture conditions, a separate experiment was conducted, measuring autohydrolysis of each DOP substrate over 20 days. P*i* concentration was measured in −P*i* SN media amended with a single DOP compound (36 µmol l^−1^; final P concentration as in Diaz et al. 2018, 2019) and no addition of cells (Figure S1). Treatments were sampled immediately after DOP addition, and again every 5 days over a 20-day incubation period. Samples were frozen at −20°C, and P*i* [or soluble reactive phosphorus (SRP)] was measured using a standard colorimetric protocol (Hansen and Koroleff 1999) on a multimode plate reader (SpectraMax^®^ M2, Molecular Devices). Absorbance read at 880 nm was calibrated using a standard curve of monopotassium phosphate (0, 0.5, 1, 2, 5, 10, 20, 40, 50, and 75 µmol l^−1^; KH_2_PO_4_). The average detection limit of P*i* using this method, defined as three times the standard deviation of the triplicate blank measurements, was 0.125 ± 0.005 µmol l^−1^. The calibration curve was prepared with 0.2 µm filtered ALOHA seawater with a P*i* background concentration below the detection limit.

### DOP hydrolysis

The capacity of *Synechococcus* WH8102 and WH5701 to hydrolyze different DOP bond-classes was determined in the presence (whole cell) and absence (cell-free filtrate) of cells over the growth curve (Diaz et al. 2018, 2019). Some P-hydrolases are predicted to be ectoenzymes, and substantial cell-free P-hydrolase activity has been documented in diverse marine environments (Duhamel et al. 2010, Baltar et al. 2019). The inclusion of cell-free filtrate hydrolysis helped reveal the mechanisms (enzymes) involved in DOP degradation. Since the selected strains did not grow on phosphonates, only DOP compounds with P-ester and P-anhydride bonds were tested. Three substrates were selected to conduct this experiment. The short-chain 3-PolyP was tested as the representative polyphosphate substrate (P-anhydrides only). Because nucleotides ATP and AMP share the same P-ester core and contain different bond types, they were also selected. AMP was selected as the representative P-ester, while ATP, containing both P-anhydride and P-ester bonds, served as a representative with both bond-classes.

Whole cell and cell-free filtrate experiments were carried out separately. For both experiment types, *Synechococcus* strains were grown in triplicate +Pi and −Pi SN media. The +P*i* and −P*i* cultures were subsampled approximately every 3 days over ∼20 days to obtain whole cell and cell-free DOP hydrolysis results along each phase of the cellular growth curve.

To determine DOP hydrolysis rates on each subsampling day, aliquots (200 µl) of +P*i* and −P*i* treatments were amended with a single DOP substrate (3-PolyP, ATP, or AMP; 20 µmol l^−1^ P, final concentration) in triplicate wells of a nontreated standard 96-well transparent microplate and incubated over 6-h. For whole cell subsamples, aliquots were directly taken from the culture flasks, while cell-free filtrate subsamples were prepared by aseptically filtering (0.2 µm) culture aliquots before DOP hydrolysis determination. The following controls were prepared in triplicate wells of the microplate and monitored in parallel: (1) an unamended treatment (addition of cells and no DOP substrate) to track P*i* concentrations in the cultures over time, (2) a treatment amended with KH_2_PO_4_ (45 µmol l^−1^ P, final concentration) to correct for the uptake/adsorption of P*i* released from DOP by the cells (Diaz et al. 2019), and (3) boiled (15 min) filtrates amended with DOP to assess potential autohydrolysis during the 6-h incubation of the plate. P*i* concentration was measured following the colorimetric protocol described above. The unamended treatment showed negligible (below detection limit) P*i* release during the 6-h incubations for all subsamplings over the growth curve, ruling out the release of periplasmic (Kamennaya et al. 2020) and other cellular sources of P*i* as a major factor. DOP hydrolysis rates were normalized to flow cytometry cell counts to account for biomass differences between strains and treatments.

To determine if the observed hydrolysis rates were sufficient to sustain cellular P demand (Diaz et al. 2019), the following equation was used:


\begin{eqnarray*}
H = Q \times g,
\end{eqnarray*}

where *H* is the maximum hydrolysis rate (amol cell^−1^ day^−1^), *Q* is the P quota (amol cell^−1^), and *g* is the growth rate (day^−1^). P quota values were taken from the range of values reported in Bertilsson et al. (2003), Heldal et al. (2003), Fu et al. (2006), and Lopez et al. (2016) for *Synechococcus* culture isolates (inclusive of WH8102, WH8103, WH8104, and WH7803; no published values are available for WH5701) grown in +P*i* and −P*i* conditions. Specifically, the minimum and maximum P quotas for cultures grown in +P*i* (58.0 and 140.0 amol cell^−1^, respectively) and for cultures grown in −P*i* (16.0 amol cell^−1^ and 25.0 amol cell^−1^, respectively) were used. For each observed rate of hydrolysis, an expected growth rate (*g*) was calculated using the maximum hydrolysis rates (days 11–21 for WH8102, days 11–20 for WH5701) and expressed as a proportion of the observed +P*i* growth rate. Ratios greater than 1 indicate that the observed DOP hydrolysis rates were sufficient to sustain P demand similar to +P*i* (Table S2).

Comparative analysis was carried out between ATP and 3-PolyP to determine if the P-ester bond of ATP is likely degraded alongside the P-anhydride bond (Table 1). Four consecutive subsampling days, encompassing peak hydrolysis rates, were selected for each strain and media type in the whole cell experiment. As ATP contains a P-ester and two P-anhydride bonds, the substrate provides bond-class hydrolysis on a single substrate. Under complete hydrolysis of either ATP or 3-PolyP, three orthophosphates can be released; however, a maximum of two and three orthophosphates from ATP and 3-polyP, respectively, can be released by hydrolysis of P-anhydride bonds alone. Therefore, under complete P-anhydride degradation, where the P-ester bond of ATP remains intact, the ATP:3-PolyP hydrolysis ratio is 2:3 (or 66.7%) (Diaz et al. 2018). If the ATP:3-PolyP hydrolysis ratio is >66.7%, it suggests the likely degradation of the P-ester bond of ATP as well (Table 1).

**Table 1.** Comparative analysis of *Synechococcus* culture strains’ ATP and 3-PolyP hydrolysis rates. For each strain and media type, maximum hydrolysis rates for 3-PolyP and ATP were selected (days 16–23 for WH8102 and days 7–17 for WH5701). The average ratio of ATP to 3-PolyP hydrolysis (ATP:3-PolyP) is expressed as a percentage (%). P-anhydride bond degradation alone results in a hydrolysis percentage of 66.7%. A higher percentage indicates P-ester bond degradation from ATP. T scores (T; reported as absolute values) are calculated by subtracting the ATP:3-PolyP percentage from 66.7 and then dividing by the standard error (SE). The degrees of freedom (df) and two-tailed *P-*values are included.

			ATP:3-PolyP (%)				
*Synechococcus* sp.	Media type	Days	Avg	SE	T	df	*P*-value
WH8102	+P*i*	16–23	58.0	15.0	2.0	11	< .0001
	−P*i*	16–23	47.0	40.0	2.0	11	.0067
WH5701	+P*i*	7–17	37.0	26.0	4.0	11	.0102
	−P*i*	7–17	58.0	28.0	1.0	11	.0005

### Cell-free proteins

For *Synechococcus* extracellular protein identification following growth in minimal P, WH8102 and WH5701 were grown in triplicate 500 ml culture flasks in −P*i* SN media amended with 1 µmol l^−1^ KH_2_PO_4_; a concentration previously determined as being low enough to induce P-stress while still maintaining biomass (Cox and Saito 2013). In the stationary phase, each culture triplicate was transferred to an autoclaved 250 ml polypropylene bottle and centrifuged at 3200 r m^−1^ for 20 min at 4°C. The supernatant was filtered using a sterile disposable vacuum filtration system (0.2 µm). Filtrate (70 ml) was added to a prerinsed Centricon spin column (Tris Buffer; 20 mmol l^−1^, pH = 8) and centrifuged at 3200 r m^−1^ for 45 min at 4°C. The procedure was repeated three times for both strains and triplicates. The sample was then dialyzed twice with Tris buffer (20 mmol l^−1^, pH = 8), and the concentrate was recovered and brought to a final volume of ∼500 µl using Tris Buffer. A QuickStart Bradford protein assay kit (Bio-Rad), calibrated using a standard curve of gamma globulin, was used to ensure that the samples contained enough protein (minimum of 100 µg ml^−1^) for further analyses. According to the manufacturer’s instructions, a trypsin digest was carried out on 10.5 µl of each triplicate using an in-solution digestion kit (ThermoScientific). Peptide samples were analyzed at the Proteomics and Mass Spectrometry facility at the University of Georgia on a Thermo-Fisher LTQ Orbitrap Elite mass spectrometer coupled with a Proxeon Easy NanoLC system (Waltham, MA, USA) following Adams et al. (2022). Peptides were mapped to the *Synechococcus* genome (NCBI BioProject PRJNA230) (Palenik et al. 2003, McCarren et al. 2005). The protein set was searched in BLASTP against NCBI nonredundant databases, and accession numbers were cross-referenced on UniProt to confirm putative function and identify APs.

### MUF-P displacement

The affinity of *Synechococcus* APs for different DOP model substrates with varying P bond-classes was examined through its ability to inhibit the hydrolysis of the fluorogenic substrate 4-methylumbelliferyl phosphate (MUF-P) (Nedoma et al. 2007). Late-exponential phase samples of −P*i* cultures were incubated in triplicate wells of a black, nontreated 96-well microplate for each *Synechococcus* strain. Either a PolyP (3-PolyP, 45-PolyP), P-ester (ATP, AMP, Glc-6-P), or phosphonate (MPn) was added in a series of concentrations (0, 2, 5, 10, 20, 40, 70, and 100 µmol l^−1^; final P concentration) with a single concentration of MUF-P. The selected DOP substrates exhibit no apparent chemical properties conducive to irreversible binding (Reid and Wilson 1971, Holtz et al. 1999, Whisnant and Gilman 2002). The MUF-P concentration was 10% of the previously determined Michaelis–Menten constant (*K*_m_). This value was established by incubating 200 µl of each *Synechococcus* strain with a range of MUF-P concentrations (0 to 20 µmol l^−1^), following the equation:


\begin{eqnarray*}
V = \textit{Vmax} \times S/Km + S,
\end{eqnarray*}

where *S* and *V* are the concentrations of MUF-P and the MUF-P hydrolysis rates, respectively.

For each tested DOP model substrate, MUF-P hydrolysis velocity was determined fluorometrically (excitation/emission: 359/449, 4-methylumbelliferone –MUF), using a multimode plate reader (SpectraMax^®^ M2, Molecular Devices) at multiple time points over an incubation period of 24-h to ensure linearity. MUF-P hydrolysis inhibition (%) is defined as a decrease of MUF-P hydrolysis velocity over the tested range of model DOP substrate concentrations relative to the control without DOP (receiving only a single MUF-P concentration). To determine IC_50_, which corresponds to the DOP concentration that causes 50% inhibition of MUF-P, data (both MUF-P hydrolysis inhibition and DOP concentrations) were fitted with a sigmoid function as described in Nedoma et al. (2007). Because the amended MUF-P concentration was 10% of the *K*_m_ for both strains (i.e. 10% of 5 µmol l^−1^ = 0.5 µmol l^−1^), the IC_50_ value can be directly expressed as the inhibition constant *K*_i_ (Nedoma et al. 2007). As such, a DOP substrate with a low binding affinity will have a high *K*_i_, as a higher DOP concentration is required to inhibit MUF-P hydrolysis.

### Data and statistical analyses

Statistical analyses for the displacement experiment were carried out in MATLAB. DOP concentrations that inhibit MUF-P hydrolysis velocities by 50% were compared using a one-way ANOVA. Flow cytometry data were processed using FCS Express 7. All remaining data analyses were performed in Microsoft Excel and GraphPad Prism 8. Substrates were compared following a two-way ANOVA and *post hoc* testing with Dunnet’s method (between experimental and control treatments) to assess culture growth on DOP. Differences in cell-normalized hydrolysis rates were evaluated using repeated measures ANOVA and *post hoc* testing with either Tukey’s honest significant difference (between the two strains and substrates under each growth condition) or Dunnett’s (between experimental and control treatments) method.

## Results

### Growth on phosphoanhydrides, phosphoesters, and phosphonates

**Figure 1.**
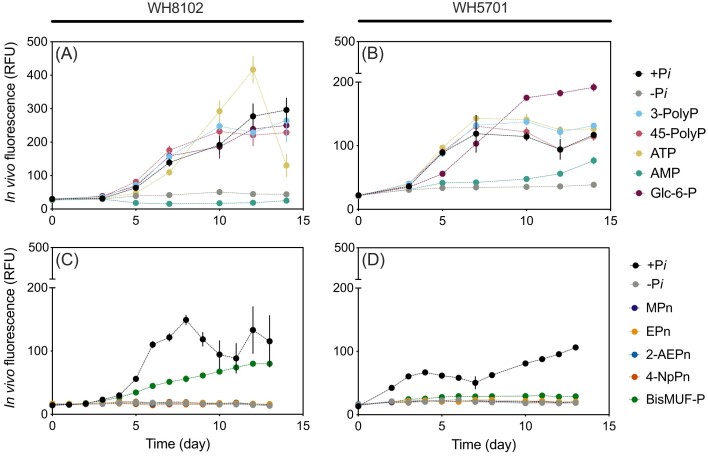
*Synechococcus* growth on DOP. *Synechococcus* WH8102 (A) and (C) and WH5701 (B) and (D) were grown on a single DOP substrate as the sole P source in two experiments, assessing IVF (RFU; *y*-axis) over time (day; *x*-axis) for P-monoesters and polyphosphates (A) and (B), as well as a P-diester and phosphonates (C) and (D). Representative DOP compounds included the P-monoesters Glc-6-P and AMP; the P-diester BisMUF-P; the short and long chain polyphosphates: 3-PolyP and 45-PolyP; the P-monoester and P-anhydride containing ATP; and the phosphonates: 4-NpPn, 2-AEPn, MPn, and EPn. Error bars indicate one standard deviation of the mean of three biological replicates. All symbols not visible at the *x*-axis showed negligible growth, not significantly different (*P* > .05) from the −P*i* treatment.

Axenic cultures of *Synechococcus* WH8102 and WH5701 grew on a variety of DOP compounds, inclusive of P-esters and P-anhydrides bond-classes, as a sole source of added P (Fig. 1, Table S1). Both strains grew on P-anhydride containing compounds 3-PolyP, 45-PolyP, and ATP with growth rates equivalent to (*P* > .05; one-way ANOVA), or greater than (*P* < .05; one-way ANOVA) the +P*i* treatment (i.e. *>* 0.5 day^−1^; Table S1). Additionally, both strains grew on the P-ester Glc-6-P similar to the +P*i* treatment (*P* < .01 for WH5701, *P* = .3814 for WH8102; one-way ANOVA). However, on the P-ester AMP, both strains exhibited growth rates significantly lower than the +P*i* control (*P* < .0001; one-way ANOVA). Specifically, for WH8102, growth on AMP was equivalent to growth on −P*i* (*P* > .05; one-way ANOVA), and despite a slight increase in IVF for WH5701 on AMP by day 14, its growth rate only reached half that on +P*i* (Fig. 1B). Neither strain grew on phosphonates MPn, EPn, 2-AEPn, and 4-NpPn, with IVF values significantly lower than the +P*i* control (*P* < .05; two-way ANOVA, Fig. 1C and D) and negligible growth rates, similar to the −P*i* treatment (< 0.15 day^−1^; Table S1). Growth on the P-diester BisMUF-P varied between strains. For WH8102, IVF values steadily increased, albeit lower than +P*i* and with a growth rate half that of +P*i* (0.20 ± 0.02 day^−1^; Table S1), while for WH5701, IVF values remained negligible over the growth curve and exhibited a growth rate (0.11 ± 0.01 day^−1^; Table S1) 3-fold lower than +P*i*, similar to −P*i*. Over the 20-day autohydrolysis experiment, DOP with P-anhydride bonds (3-PolyP, 45-PolyP, and ATP) were autohydrolyzed. Low abiotic degradation occurred in the initial 5 days; by day 20, 19.0 ± 1.5 µmol l^−1^ of 3-PolyP, 9.0 ± 1.0 µmol l^−1^ of ATP, and 7.0 ± 6.0 µmol l^−1^ of 45-PolyP were abiotically degraded (Figure S1). However, the rates of abiotic degradation were negligible compared to the whole cell DOP hydrolysis rates measured over 6-h (see below).

### DOP hydrolysis

**Figure 2.**
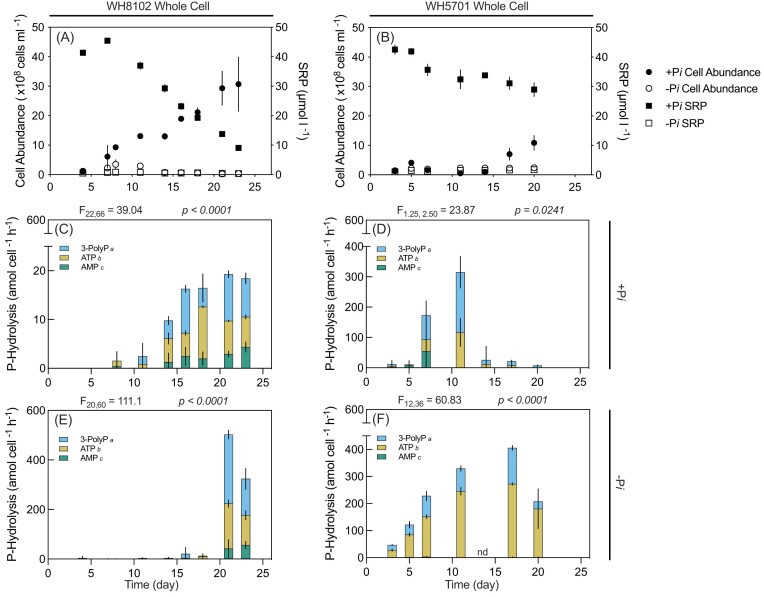
*Synechococcus* DOP hydrolysis. SRP concentrations (µmol l^−1^; squares) and cell abundance (×10^8^ cells ml^−1^; circles) in P*i*-replete (+P*i*; filled symbols) and P*i*-deplete (−P*i*; empty symbols) media are displayed over time (day) for WH8102 (A) and WH5701 (B). P-hydrolysis rates on selected model DOP substrates (3-PolyP, ATP, and AMP; amol cell^−1^ h^−1^) in +P*i* (C) and (D) and −P*i* (E) and (F) are normalized to cell abundance. P-hydrolysis rates are represented as overlapping bars. Error bars indicate one standard deviation of the mean of biological triplicates. Statistical results from repeated measures ANOVA are provided above each P hydrolysis plot (C)–(F), indicating the significance of DOP hydrolysis rates throughout the experiment. Results from the pairwise *post hoc* comparison of each DOP source *via* Tukey’s honest significant difference test are provided next to the legend entries. DOP sources lacking a shared letter differ significantly (*P* < .05). Days in which hydrolysis rates were not collected are denoted as “no data” (nd).

To assess P-ester and P-anhydride degradation, hydrolysis rates of a representative P-anhydride (3-PolyP) and P-ester (AMP), as well as a DOP compound with both P-anhydride and P-ester bonds (ATP), were measured for *Synechococcus* WH8102 and WH5701 grown in +P*i* and −P*i* SN media (Fig. 2). All tested DOP substrates showed autohydrolysis below the detection limit over the 6-h incubation for the whole cell and cell-free filtrate experiments. In the whole cell experiment, hydrolysis rates per cell generally increased with time over the growth curve, consistent with the decrease in media P*i* concentration. Higher per-cell hydrolysis rates were observed in the −P*i* treatment relative to +P*i* (*P* < .05; one-way ANOVA; Fig. 2). Specifically, for WH8102, maximum hydrolysis rates for 3-PolyP, ATP, and AMP were ∼24-fold higher in the −P*i* treatment than in the +P*i* treatment. For WH5701, maximum hydrolysis rates in the −P*i* treatment were 32.0 ± 14.0-fold higher for 3-PolyP, 2.5 ± 1.0-fold higher for ATP, and 3.0 ± 2.0-fold higher for AMP, in comparison to the +P*i* treatment. Regardless of strain and P*i* availability, throughout the experiments, 3-PolyP hydrolysis rates were significantly higher than ATP, and ATP hydrolysis rates were significantly higher than AMP (*P* < .05; Fig. 2C–F). Maximum DOP hydrolysis rates were observed for 3-PolyP in the −P*i* treatment at the onset of the stationary phase, reaching 502.0 ± 17.0 amol cell^−1^ h^−1^ for WH8102 and 406.0 ± 8.5 amol cell^−1^ h^−1^ for WH5701.

In the final 2 weeks, corresponding to the highest observed DOP hydrolysis rates (Diaz et al. 2018), the ATP:3-PolyP hydrolysis ratio was less than 2:3, or 66.7%, for both strains and P*i* treatments. This result suggests that the P-ester bond of ATP remained intact while the P-anhydride bond was hydrolyzed (Table 1). In the early phase of the growth curve, AMP hydrolysis rates were below detection in both strains and P*i* treatments. By day 23 for WH8102 grown on +P*i*, low but consistent hydrolysis rates were observed (4 ± 1 amol cell^−1^ h^−1^; Fig. 2C). For the remaining strains and media types, while AMP hydrolysis was observable on certain individual sampling days, rates did not increase over time (*P* > .05; two-way ANOVA; Fig. 2D–F).

DOP hydrolysis activity was verified in the cell-free filtrate (Figure S2), indicating the presence of cell-free P-hydrolase enzymes. DOP hydrolysis rates in −P*i* filtrates were observed over the growth curve (*P* < .001; one-way ANOVA; Figure S2E and F), with 3-PolyP hydrolysis rates significantly higher than ATP hydrolysis rates (Figure S2E and F). AMP hydrolysis rates were negligible in all filtrates. Maximum hydrolysis rates observed in the filtrates occurred for 3-PolyP in the −P*i* treatment on day 21 (471.0 ± 11.0 amol cell^−1^ h^−1^ for WH8102, 220.0 ± 1.0 amol cell^−1^ h^−1^ for WH5701; Figure S2E and F). DOP hydrolysis by WH8102 grown in +P*i* media was minimal over the growth curve, with no significant difference over time and between treatments (*P =* .6250; Figure S2C). Hydrolysis rates were minimal for WH5701 grown in +P*i* media, except on day 21 (64.0 ± 3.0 amol cell^−1^ h^−1^ for 3-PolyP, 23.0 ± 1.0 amol cell^−1^ h^−1^ for ATP, 1.0 ± 0.5 amol cell^−1^ h^−1^ for AMP; Figure S2D).

### Cell-free proteins

Overall, 43 and 25 proteins were present in the exoproteome of P*i*-limited WH8102 and WH5701, respectively. Of that, 30% for WH8102 and 16% for WH5701 were hypothetical unidentified proteins (Tables S3 and S4). Among the annotated proteins were ones associated with phosphate acquisition pathways. A phosphate ABC transporter was identified in WH8102 (CAE07533.1) and WH5701 (EAQ75702.1). For WH8102, putative APs (CAE08906.1 and CAE8314.1), phosphorylase (CAE06671), and nucleoside diphosphate kinase (CAE08873.1) were present, though not across all replicates (replicates 2–6, replicates 2 and 5, replicates 4–6, and replicates 2, 4–6; respectively). Among the identified porins, somSYNW2224 (CAE08739.1) was previously shown to be upregulated under P*i* stress. For WH5701, a putative AP (EAQ75607.1) and protein phosphatase 2C (EAQ76317.1) were the only (alongside the ABC transporter) proteins present with a known association with phosphate acquisition pathways.

### MUF-P displacement

**Figure 3.**
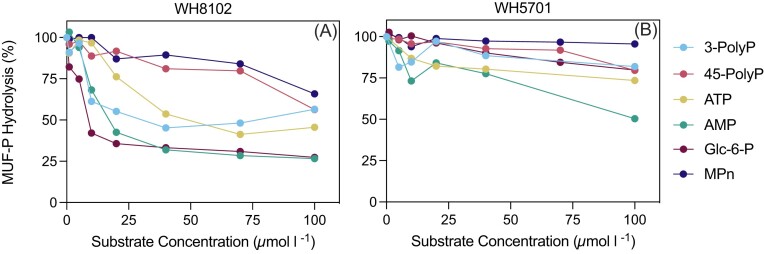
*Synechococcus* MUF-P hydrolysis inhibition by DOP. DOP substrates were applied at six different concentrations (*x*-axis; 0–100 µmol l^−1^) in the presence of 4-methylumbelliferyl phosphate (0.5 µmol l^−1^; MUF-P) for P*i*-deplete *Synechococcus* WH8102 (A) and WH5701 (B). MUF-P hydrolysis, represented as a percentage (%) of the control (no DOP addition), is displayed on the *y*-axis. Dots represent the average of three biological replicates, and error bars are omitted for visual clarity. Biological triplicates typically agreed to within ± 9%.

To test substrate specificities of *Synechococcus* APs across bond-classes, AP activity was measured using the fluorogenic substrate MUF-P with and without the addition of competing DOP substrates in P*i*-deplete cultures (Fig. 3). Specifically, P-anydrides (3-PolyP and 45-PolyP), P-esters (Glc-6-P and AMP), a substrate with both P-anhydride and P-esters (ATP), and a phosponate (MPn) were tested. All DOP substrates resulted in a concentration-dependent decrease of MUF-P hydrolysis by WH8102 (Fig. 3A). At a DOP addition of 70 µmol l^−1^, Glc-6-P, AMP, and ATP resulted in a reduction in MUF-P hydrolysis ranging between 58% and 71%, followed by 3-PolyP at ∼50%, and finally 45-PolyP and MPn at ∼20%. In comparison, the response of WH5701 was generally muted; by 100 µmol l^−1^ DOP additions, MUF-P hydrolysis decreased by ∼45% for AMP, ∼20% for Glc-6-P, 3-PolyP, 45-PolyP, and ATP, and ∼10% for MPn (Fig. 3B). The inhibition constant, K*_i_*, which corresponds to the DOP substrate concentration necessary to inhibit MUF-P hydrolysis by 50%, reflects the binding affinity (the smaller the K*_i_*, the greater the binding affinity; Table 2). For WH8102, K*_i_* was lowest for Glc-6-P, AMP, and 3-PolyP (∼7–11 µmol l^−1^), then ATP (∼24 µmol l^−1^) and could not be calculated for long-chain 45-PolyP and MPn. For WH5701, K*_i_* values could not be calculated for short and long-chain PolyP, as well as MPn and AMP, and required high concentrations of Glc-6-P and ATP (117–125 µmol l^−1^) addition to inhibit MUF-P hydrolysis by 50% (Table 2).

**Table 2.** Inhibition constants for the displacement of MUF-P by DOP. Inhibition constants (K_*i*_), which correspond to the concentrations necessary for half saturation of phosphatases by different DOP substrates, are presented for *Synechococcus* strains WH8102 and WH5701. DOP substrates included short and long chain polyphosphates: 3-PolyP and 45-PolyP; the P-monoester and P-anhydride containing adenosine 5′-triphosphate (ATP); the P-monoesters AMP and Glc-6-P; and phosphonate MPn. Treatments in which the DOP addition did not result in a 50% (at minimum) 4-methylumbelliferyl phosphate (MUF-P) inhibition are denoted as “not applicable” (n.a.).

K*_i_* constant (µmol l^−1^)						
	3-PolyP	45-PolyP	ATP	AMP	Glc-6-P	MPn
**WH8102**	13 ± 4	n.a.	24 ± 5	11 ± 3	7 ± 3	n.a.
**WH5701**	n.a.	n.a.	125 ± 37	n.a.	117 ± 12	n.a.

## Discussion

The primary objective of this study was to assess the bond-specific bioavailability and utilization of DOP compounds to *Synechococcus*. For both the open ocean strain (WH8102) and the coastal strain (WH5701), our results indicate a preference for the enzymatic hydrolysis of DOP compounds containing P-anhydride bonds (i.e. 3-PolyP, 45-PolyP, and ATP).

### Growth on phosphoanhydrides, phosphoesters, and phosphonates

*Synechococcus* is a widespread cyanobacterium that is frequently exposed to low P*i* concentrations, suggesting the need for a mechanism to cope with P*i* scarcity (Lomas et al. 2010, Duhamel et al. 2014, 2021, Karl and Björkman 2015). DOP can serve as a P source in addition to P*i*, though the DOP pool contains a range of bond types of unknown bioavailability for the microbial community (Karl and Björkman 2015, Hull and Ruttenberg 2022). Although it is widely believed that P-esters play a predominant role in microbial interactions with DOP (i.e. Karl and Björkman 2015), our results indicate that *Synechococcus* consistently exhibits growth on DOP substrates containing P-anhydrides, shows negligible growth on phosphonates, and displays substrate-dependent growth on P-esters.

PolyP only accounts for ∼8%–13% of high molecular weight dissolved organic matter (Young and Ingall 2010, Diaz et al. 2016, Saad et al. 2016), and 1%–5% of the DOP pool in coastal environments (Bell et al. 2017, 2020). That said, both the open ocean and coastal *Synechococcus* strains exhibited robust growth when cultured with P-anhydride containing compounds (specifically, 3-PolyP, 45-PolyP, and ATP), comparable to their growth with P*i* as the sole P source (Fig. 1). These results indicate that the selected P-anhydride compounds are bioavailable to both strains, which builds on prior results for WH8102 (Moore et al. 2005). While phosphonate utilization pathways, such as transport genes (*phnDCE*), have been previously identified for *Synechococcus* (Moore et al. 2005, Ilikchyan et al. 2009, Shah et al. 2023), neither strain grew on phosphonates as a sole P source. Similarly, Shah et al. (2023) recently identified a lack of WH8102 growth on methylphosphonate, prompting the reinterpretation of its *phnDCE* genes as regulatory factors in P*i* transport.

Phosphoesters, including diesters and monoesters, comprise most of the high molecular weight dissolved organic matter (∼80%–85%; Young and Ingall 2010), as well as coastal environment DOP pool (∼60% and ∼30%, respectively; Bell et al. 2020). Phosphodiesterase and phosphomonoesterase activity in marine environments suggest that P-diesters are less bioavailable than P-monoesters (Sato et al. 2013). Here, *Synechococcus* strains exhibited varying growth patterns when provided the P-diester BisMUF-P. WH8102 displayed an initially stunted growth curve that eventually increased, reaching maximum IVF values similar to P*i*. In contrast, WH5701 demonstrated minimal growth on BisMUF-P (Fig. 1) These results imply that P-diesters may be more bioavailable to strains from the open ocean, where P*i* is frequently limited or colimited, compared to coastal environments (Moore et al. 2013, Browning and Moore 2023). The delayed growth also suggests that for the open ocean strain, consistent and prolonged exposure to P-diesters may be required for the culture to employ a mechanism for utilization. This could include producing phosphodiesterases or AP that can hydrolyze P-monoesters and P-diesters (Srivastava et al. 2021).

*Synechococcus* strains displayed diverse growth responses when exposed to P-monoesters. Both strains grew on Glc-6-P similar to the +P*i* treatment (Table S1); a result that aligns with *Synechococcus* WH7803 P*i* cleavage of Glc-6-P (Donald et al. 1997). However, WH8102 failed to grow on AMP, and although WH5701 showed slight growth on AMP, it was delayed, and far less than the +P*i* treatment (Fig. 1). This is in agreement with the low and undetectable rates of AMP hydrolysis in cultures grown in +P*i* or −P*i* conditions (Fig. 2). The delayed growth on AMP implies that similar to P-diester BisMUF-P, the culture may require prolonged and consistent exposure to AMP for sustained growth. It is possible that WH5701 could utilize AMP *via* direct uptake and/or low (undetectable) rates of enzymatic hydrolysis. However, for *E. coli*, enzymatic cleavage of AMP by 5′-nucleotidase (5′-NT) is necessary for subsequent uptake of P*i* (Yagil and Beacham 1975), and to our knowledge, there is no evidence for AMP direct uptake by *Synechococcus*. Although 5′-nucleotidase is present in the *Synechococcus* genome, it was not identified in either strain in the exoproteome analysis. Overall, these results suggest that different P-esters exhibit varying levels of bioavailability, emphasizing the importance of substrate diversity and complexity of P cycling in marine environments.

### DOP hydrolysis fulfilling P demand

In agreement with their growth on P-anhydride substrates as a sole P source, both −P*i* strains hydrolyzed 3-PolyP and ATP. Hydrolysis rates of 3-PolyP and ATP consistently and largely exceeded P demand for growth, in both strains grown in +P*i* or −P*i* media, and assuming a range of P quotas (Table S2). For WH8102 in +P*i*, AMP hydrolysis was sufficient in meeting the P demand only if the minimum P quota was assumed, while for WH5701 in +P*i*, hydrolysis rates were insufficient regardless of the P quota. AMP hydrolysis was not detectable for WH8102 and WH5701 in −P*i* until day 21 and 20, respectively, at which point, despite being substantially lower than 3-PolyP and ATP hydrolysis, met the P demand. Culture growth on DOP substrates was only monitored for 14 days, at which point cultures on +P*i* and DOP, supporting equal growth, had reached the stationary phase for several days. Therefore, it is possible that longer incubations would have been necessary to observe sustained growth of both strains on AMP.

DOP hydrolysis was observed over the growth curve even in the +P*i* media with background P*i* concentrations at 34.0 ± 1.0 µmol l^−1^ for WH5701 and 29.0 ± 1.5 µmol l^−1^ for WH8102 by day 14; Fig. 2A and B). Previous studies documented DOP hydrolysis in natural communities under relatively high P*i* concentrations (Benitez-Nelson and Buesseler 1999, Nausch et al. 2018). The measurable release of P*i* into the media indicates a lack of strict coupling between DOP hydrolysis and P*i* uptake. These combined results suggest that *Synechococcus* P-hydrolase enzymes, whether cell-free or cell-associated, liberate P*i* into their environment, potentially offering a source for assimilation by other microbes. These results are consistent with the high AP activity measured across diverse natural environments with different P*i* concentrations (Duhamel et al. 2011, Thomson et al. 2019). Given that growth rates on the three P-anhydride substrates either equaled or surpassed those on the +P*i* control and considering that the hydrolysis of DOP containing P-anhydride bonds exceeded the estimated P growth demand, these combined results strongly support the capability of both strains to thrive on P-anhydride compounds.

### Preferential hydrolysis of the phosphoanhydride bond

Across strains and P*i* availability, 3-PolyP hydrolysis rates were higher than ATP hydrolysis rates, and both were higher than AMP hydrolysis rates, confirming the preferential hydrolysis of the P-anhydride bond. Comparative analysis between ATP and 3-PolyP aims to assess whether the P-ester bond of ATP degrades alongside the P-anhydride bond (Diaz et al. 2018). For each strain and media type, the ATP:3-PolyP hydrolysis ratio consistently remained below 2:3. This observation suggests that the P-anhydride bond of ATP was degraded while the P-monoester bond of ATP remained intact. Similar results have been reported in the case of the open ocean diatom *Thalassiosira* sp. CCMP1005 and CCMP1014, as well as coastal *Thalassiosira* sp. CCMP 1335 (Diaz et al. 2018). These consistent patterns underscore the potential significance of the P-anhydride bond specificity in microbial DOP bioavailability.

### Cell-free filtrate P-Hydrolases

Marine plankton can obtain P from the hydrolysis of DOP using their cell-surface-associated enzymes (Cembella et al. 1984, Ammerman and Azam 1985). Some of these enzymes can also be liberated from the cell by secretion or upon death (cell lysis or sloppy feeding), which contributes to the cell-free P-hydrolase activity measured across P*i*-replete and P*i*-deplete marine environments (Duhamel et al. 2010, Baltar et al. 2019). Using cell-free culture filtrates from both strains of *Synechococcus*, we found negligible hydrolysis of the three tested DOP substrates under +P*i* treatment but high hydrolysis rates of 3-PolyP and ATP in the −P*i* cultures (Figure S2), confirming the presence of enzymes that act on DOP. While the cell-free filtrate is expected to include a mixture of naturally and artificially released extracellular enzymes (i.e. via filtration), this method allows us to narrow down the enzymes potentially involved in DOP hydrolysis.

Both *Synechococcus* strains hydrolyzed PolyP in the whole cell and cell-free experiments, suggesting the presence of an enzyme that can specifically hydrolyze P-anhydrides. The exoproteome of P*i*-depleted strains revealed AP isoforms phoX and phoA (for WH8102) and unidentified AP (for WH5701; accession EAQ75607.1). The previously reported genome of both strains contain enzymes known for acting on PolyP and P-esters, including AP isoforms phoA and phoX; and 5′-nucleotidase (5′-NT) and polyphosphatase (ppX) (Moore et al. 2005, Scanlan et al. 2009, Tetu et al. 2009, Kutovaya et al. 2013, Christie-Oleza et al. 2015). Our results did not have PolyP-specific enzymes, so the observed extracellular PolyP hydrolysis is likely not dominated by PolyP-specific enzymes. This result aligns with the outcome of Adams et al. (2022). It does not exclude the possibility of direct low molecular weight PolyP uptake followed by interactions with ppK (synthesis and degradation) (Parnell et al. 2018) and ppX (degradation) or the potential for a low biomass sample (resulting from growth on minimal P*i*) to impact the detection of additional enzymes in our samples.

### AP flexibility

Following the presence of APs in both *Synechococcus* strains, matched with 3-PolyP and ATP hydrolysis and the absence of PolyP specific enzymes, it is likely that APs play a role in the hydrolysis of P-esters and PolyP in *Synechococcus*. Though it is generally assumed that APs only hydrolyze P-monoesters (Tiwari et al. 2015), there is increasing evidence of AP substrate flexibility. APs from *E. coli* and calf intestine can cleave P-anhydride bonds (Yoza et al. 1997, Huang et al. 2018). Long-chain PolyP substrates, up to 800 monomers, may be cleaved by mammalian AP depending on the concentration and ambient pH levels, indicating that AP substrate specificity may vary (Lorenz and Schröder 2001). To test this, we determined the ability of P-ester, P-anhydride, and phosphonate substrates to compete with MUF-P for the reaction of APs from *Synechoccocus* (Fig. 3, Table 2).

The MUF-P displacement experiments provide insight into the relative affinity of AP for the DOP bond-classes. All tested substrates inhibited MUF-P hydrolysis by WH8102, indicating that *Synechococcus* WH8102 APs have broad substrate specificities (Fig. 3, Table 2), similar to results reported from the bacterial copiotroph *R. pomeroyi* (Adams et al. 2022). Specifically, P-esters Glc-6-P and AMP exhibited the highest MUF-P inhibition, followed by ATP and short-chain 3-PolyP. Long-chain 45-PolyP and MPn also resulted in a decrease in MUF-P hydrolysis. However, 100 µmol l^−1^ of each substrate was required to reach the result of other P-anhydrides at 30 µmol l^−1^, suggesting a low AP-binding affinity for both long-chain polyphosphate and phosphonate MPn. These results suggest a short-chain PolyP preference by *Synechococcus* WH8102 APs. This contradicts the results of *R. pomeroyi* (Adams et al. 2022), possibly indicating different AP flexibilities across microbial groups.

For WH5701, the response to DOP was muted, suggesting a lower AP affinity for the selected DOP substrates in the tested concentration range. At high DOP concentrations (100 µmol l^−1^), P-ester and PolyP substrates decreased MUF-P hydrolysis, though less than 50%, while phosphonate MPn did not. Since we measured high hydrolysis rates of ATP and 3-polyP via P*i* production assays, the overall low AP affinity for these substrates suggests that WH5701 likely produces enzymes that can hydrolyze PolyP and P-esters better than synthetic MUF-P. This result also highlights the importance of studying multiple strains within *Synechococcus* when considering enzymatic responses.

## Conclusion

There is growing evidence that phosphoanhydrides, in particular PolyP, play a pivotal role in the bioavailable pool of marine P (Martin et al. 2014, Diaz et al. 2018, 2019, Li et al. 2019, Sanz-Luque et al. 2020). PolyP emerges as a crucial nutritional P source, particularly under P*i* deficiency, supporting the growth of eukaryotic phytoplankton (Diaz et al. 2018, 2019), heterotrophic bacteria (Adams et al. 2022), and *Synechococcus* WH8102 (Moore et al. 2005). This study further elucidates the importance of PolyP in open-ocean and coastal strains of *Synechococcus* and characterizes the potential for AP to drive its bioavailability and utilization. Our findings echo previous observations of the preferential degradation of P-anhydrides over P-esters, a phenomenon observed in diatoms of the genus *Thalassiosira* (Diaz et al. 2018, 2019), thus suggesting that P-anhydrides may substantially contribute to the nutritional DOP demand of phytoplankton. The relatively low concentration of P-anhydride containing compounds in natural marine DOP standing stocks could be explained by rapid microbial cycling (Young and Ingall 2010, Martin et al. 2014, Diaz et al. 2016, Bell et al. 2017, 2020). While AP activity has traditionally been a measure of P-ester hydrolysis, our study underscores its substantial substrate flexibility, including for short-chain P-anhydride hydrolysis. Notably, this flexibility varies between the *Synechococcus* WH8102 and WH5701 strains. A comprehensive understanding of DOP bioavailability necessitates the characterization of the enzymes involved. This study emphasizes the importance of further characterizing these enzymes, including AP flexibility for different DOP bond-classes and within taxonomic groups. These insights contribute to our understanding of marine nutrient cycling and have implications for ecosystem dynamics and biogeochemical processes.

## Supplementary Material

fiae099_Supplemental_Files
