# Supplementary material for: Dissolved organic phosphorus bond-class utilization by Synechococcus
Source: FEMS Microbiol Ecol. 2024 Jul 13;100(9):fiae099. doi: 10.1093/femsec/fiae099 (PMC11319936; doi:10.1093/femsec/fiae099)
Supplement: fiae099_Supplemental_Files [file fiae099_supplemental_files.zip › Supplementary_Data_Waggoner_FEMS.docx]

Supplementary Data

Dissolved Organic Phosphorus Bond-Class Utilization by *Synechococcus*

Emily M. Waggoner^1^, Kahina Djaoudi^1^, Julia M. Diaz^2^, Solange Duhamel^1*^

^1^Department of Molecular and Cellular Biology, University of Arizona, Tucson, AZ, US

^2^Geosciences Research Division, Scripps Institution of Oceanography, University of

California, San Diego, La Jolla, CA, US

**Keywords:** dissolved organic phosphorus_1_, alkaline phosphatase_2_, phosphoester_3_,

Phosphoanhydride_4_, Synechococcus_5_, phosphonate_6_

^*^ Corresponding author:

Solange Duhamel

University of Arizona, Life Sciences South 354

1007 East Lowell Street

Tucson, Arizona, US 85721

duhamel@arizona.edu

**
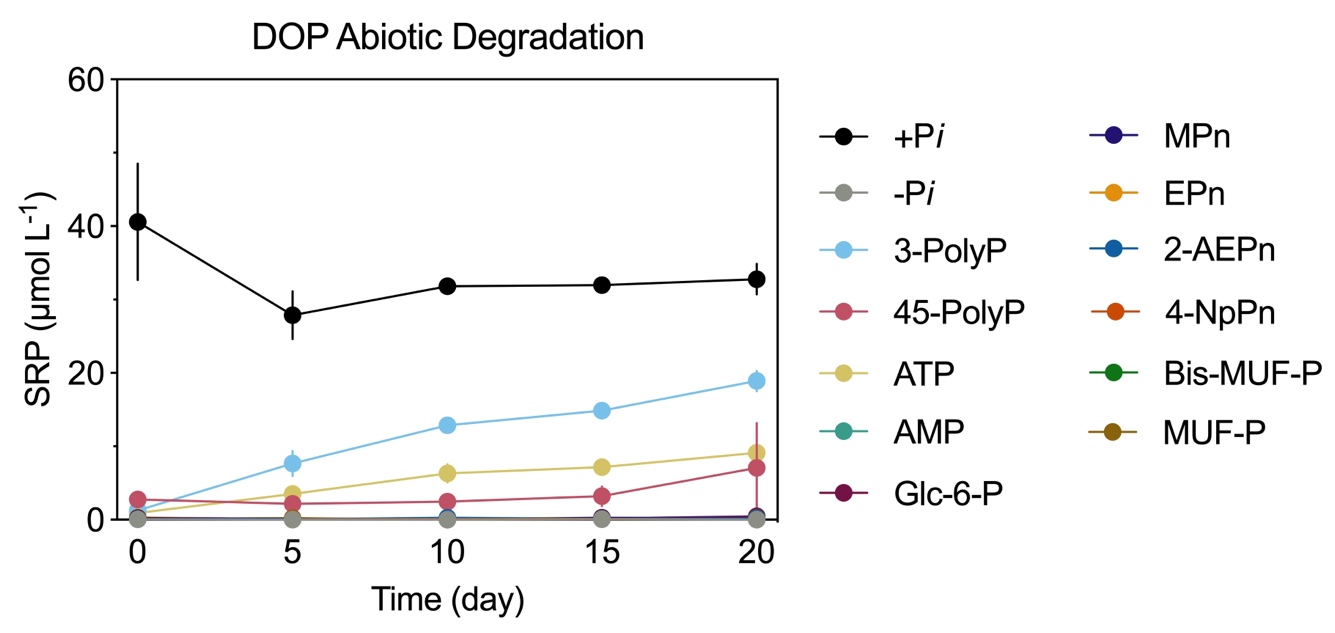
**

**Supplementary Figure 1: DOP abiotic degradation.** A single DOP substrate was added to -P*i* SN media without the addition of cells. Soluble reactive phosphorus (SRP) concentrations (µmol L^-1^), or a measure of P*i* production through hydrolysis, are displayed on the y-axis. Error bars indicate one standard deviation of the mean of three biological replicates. All symbols not visible at the x-axis showed negligible SRP, not significantly different (*p < 0.05*) from the -P*i* treatment.


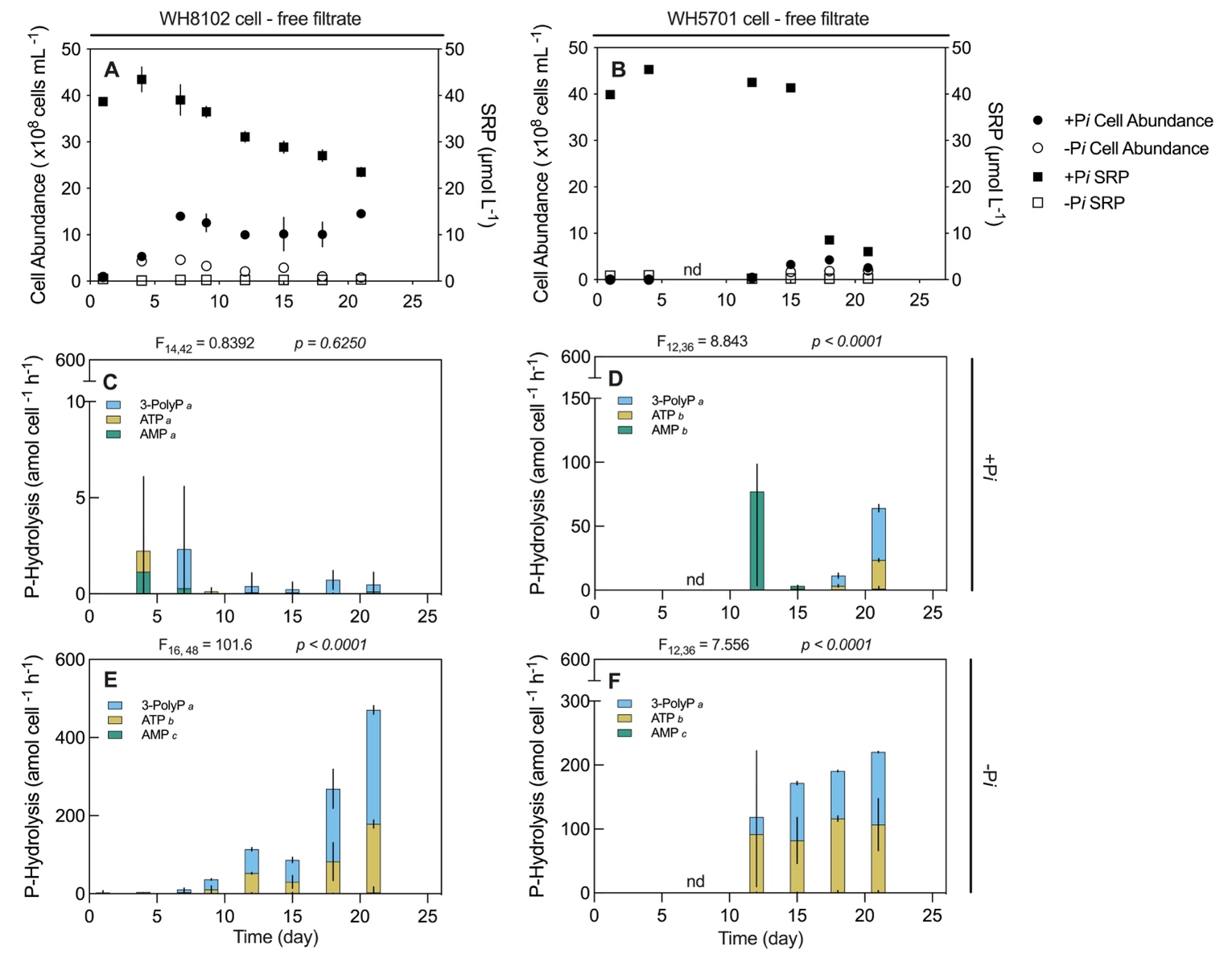


**Supplementary Figure 2: *Synechococcus* cell-free filtrate DOP hydrolysis.** Soluble reactive phosphorus (SRP) concentrations (µmol L^-1^; squares) and cell abundance (x10^8^ cells mL^-1^; circles) in P*i*-replete (+P*i*; filled symbols) and P*i*-deplete (-P*i*; empty symbols) media are displayed over time (day) for WH8102 (A) and WH5701 (B). P-hydrolysis rates on selected model DOP substrates (3-PolyP, ATP, and AMP; amol cell^-1^ h^-1^) in +P*i* (C, D) and -P*i* (E, F) are normalized to cell abundance. P-hydrolysis rates are represented as overlapping bars. Error bars indicate one standard deviation of the mean of three biological replicates. Statistical results from repeated measures ANOVA are provided above each P-hydrolysis plot (C–F) and indicate the significance of DOP hydrolysis throughout the experiment. Results from the pairwise post hoc comparison of each DOP source via Tukey's honest significant difference test are provided next to the legend entries. DOP sources lacking a shared letter differ significantly (*p < 0.05*). Days in which hydrolysis rates were not collected are denoted as "no data" (nd).

**Supplementary Table 1: *Synechococcus* growth rates on DOP.** *Synechococcus* WH8102 and WH5701 were grown on a single DOP substrate as the sole phosphorus source in two experiments: one for P-monoesters and polyphosphates (A), and one for a P-diester and phosphonates (B). Growth rates (day^-1^) were calculated as the slope of the best-fit line over the natural log-linear portion of the *in vivo* fluorescence (Figure 1) and represent the average of three biological replicates (± SD). Representative DOP compounds included the P-monoesters glucose-6-phosphate (Glc-6-P) and adenosine 5’-monophosphate (AMP); the P-diester Bis(4-methylumbelliferyl) phosphate (BisMUF-P); the short and long chain polyphosphates: 3-polyphosphate (3-PolyP) and 45-polyphosphate (45-PolyP); the P-monoester and P-anhydride containing adenosine 5’-triphosphate (ATP); and the phosphonates: 4-nitrophenyl phenylphosphonate (4-NpPn), 2-aminomethylphosphonic acid (2-AEPn), methylphosphonic acid (MPn), and ethylphosphonic acid (EPn)*.*

1. Growth Experiment on P-monoesters and Polyphosphates

| **Phosphorus Source** | ***Synechococcus* WH8102**  **Growth Rate (day^-1^)** | ***Synechococcus* WH5701**  **Growth Rate (day^-1^)** |
| --- | --- | --- |
| +P*i* | 0.54 ± 0.04 | 0.59 ± 0.08 |
| -P*i* | 0.17 ± 0.01 | 0.04 ± 0.01 |
| 3-PolyP | 0.66 ± 0.03 | 0.62 ± 0.0 |
| 45-PolyP | 0.63 ± 0.02 | 0.63 ± 0.01 |
| ATP | 0.69 ± 0.02 | 0.66 ± 0.01 |
| AMP | 0.15 ± 0.03 | 0.26 ± 0.06 |
| Glc-6-P | 0.59 ± 0.01 | 0.49 ± 0.01 |

1. Growth Experiment on a P-diester and Phosphonates

| **Phosphorus Source** | ***Synechococcus* WH8102**  **Growth Rate (day^-1^)** | ***Synechococcus* WH5701**  **Growth Rate (day^-1^)** |
| --- | --- | --- |
| +P*i* | 0.47 ± 0.02 | 0.36 ± 0.06 |
| -P*i* | 0.04 ± 0.0 | 0.07 ± 0.02 |
| BisMUF-P | 0.20 ± 0.02 | 0.11 ± 0.01 |
| MPn | 0.06 ± 0.01 | 0.13 ± 0.03 |
| EPn | 0.05 ± 0.01 | 0.12 ± 0.06 |
| 2-AEPn | 0.05 ± 0.01 | 0.03 ± 0.01 |
| 4-NpPn | 0.04 ± 0.02 | 0.09 ± 0.03 |

**Supplementary Table 2: *Synechococcus* maximum DOP hydrolysis rates, expected growth rates, and proportion to growth demand.** The expected growth rates were calculated using the phosphorus (P) quota (amol cell^-1^) and maximum hydrolysis rates (amol cell^-1^ day^-1^). The P quota included the minimum and maximum (lines 1 and 2, respectively) P quotas of each media type (58 and 140 amol cell^-1^ for +P*i*, 16 and 25 amol cell^-1^ for -P*i*). Maximum hydrolysis rates were selected as days 11–21 for WH8102 and 11–20 for WH5701. Maximum hydrolysis rates and the expected growth rates are listed as the average (± SD) of three biological replicates. AMP hydrolysis in the -P*i* media was undetectable until day 21 for WH8102 and 20 for WH5701, resulting in high standard deviations. The expected growth rates were compared to the actual +P*i* growth rates to determine the proportion to growth demand. Values greater than 1 indicate hydrolysis rates that are enough to sustain culture P demand similar to +P*i*.

| ***Synechococcus* Strain** | **Substrate** | **Maximum Hydrolysis**  **(amol cell^-1^ day^-1^)** | **P Quota Range**  **(amol cell^-1^)** | **Expected Growth Rate (day^-1^)** | **Proportion to Growth Demand** |
| --- | --- | --- | --- | --- | --- |
| WH8102 +P*i* | 3-PolyP | 309.0 ± 30.0 | 58.0  140.0 | 5.3 ± 0.5  2.2 ± 0.2 | 10.30  4.30 |
|  | ATP | 175.0 ± 5.0 | 58.0  140.0 | 3.0 ± 0.1  1.3 ± 0.0 | 5.85  2.40 |
|  | AMP | 41.5 ± 11.0 | 58.0  140.0 | 0.7 ± 0.2  0.3 ± 0.1 | 1.40  0.60 |
| WH8102 -P*i* | 3-PolyP | 2595.0 ± 105.0 | 16.0  25.0 | 162.0 ± 6.5  104.0 ± 4.2 | 314.00  201.00 |
|  | ATP | 1183.0 ± 48.0 | 16.0  25.0 | 74.0 ± 3.0  47.0 ± 2.0 | 143.10  91.60 |
|  | AMP | 212.0 ± 187.0 | 16.0  25.0 | 13.3 ± 11.7  8.5 ± 7.5 | 25.70  16.45 |
| WH5701 +P*i* | 3-PolyP | 2277.0 ± 81.0 | 58.0  140.0 | 38.0 ± 1.4  15.8 ± 0.6 | 78.05  32.30 |
|  | ATP | 813.0 ± 245.0 | 58.0  140.0 | 14.0 ± 4.2  5.8 ± 1.7 | 28.65  11.85 |
|  | AMP | 2.0 ± 0.5 | 58.0  140.0 | 0.03 ± 0.00  0.02 ± 0.00 | 0.07  0.03 |
| WH5701 -P*i* | 3-PolyP | 5662.0 ± 14.0 | 16.0  25.0 | 354.0 ± 0.9  226.5 ± 0.5 | 722.55  462.50 |
|  | ATP | 4206.0 ± 494.0 | 16.0  25.0 | 263.0 ± 31.0  168.0 ± 20 | 536.80  343.50 |
|  | AMP | 26.0 ± 20.0 | 16.0  25.0 | 0.8 ± 0.7  1.0 ± 0.8 | 1.70  2.05 |
